# A 6.3 Mb maternally derived microduplication of 20p13p12.2 in a fetus with Brachydactyly type D and related literature review

**DOI:** 10.1186/s13039-022-00584-3

**Published:** 2022-02-28

**Authors:** Guangquan Chen, Shiyi Xiong, Gang Zou, Fengyu Wu, Xiaoxing Qu, Salem Alawbathani, Luming Sun

**Affiliations:** 1 Shanghai Key Laboratory of Maternal Fetal Medicine, Department of Fetal Medicine and Prenatal Diagnosis Center, Shanghai First Maternity and Infant Hospital, School of Medicine, Tongji University, 2699# West Gaoke road, Pudong District, Shanghai, 200092 P. R. China; 2 CENTOGENE GmbH, 18055 Rostock, Germany

**Keywords:** Prenatal diagnosis, Chromosomal microarray analysis, Trio exome sequencing, Brachydactyly, *BMP2*

## Abstract

**Background:**

With the introduction of genetic tests such as chromosomal microarray analysis (CMA) and exome sequencing (ES) into fetal medical practices, genotype–phenotype correlations in intrauterine-onset disorders have substantially improved. The *BMP2* gene, located on the long arm of chromosome 20 plays a role in bone and cartilage development and is associated with Brachydactyly type A2, an autosomal dominant disease characterized by malformations of the middle phalanx of the index finger and abnormalities of the second toe. However, the *BMP2* gene has so far never been reported as a candidate gene for Brachydactyly type D (BDD) affecting only the thumbs.

**Methods and results:**

Here, we report one family possessing a maternally inherited 6.3 Mb microduplication of 20p13p12.2 including the *BMP2* gene with discordant phenotypes between the mother and the fetus. The mother was affected with BDD alongside mild facial dysmorphism and learning difficulties, while the female fetus showed BDD, severe symmetric intrauterine growth restriction combined with oligohydramnios. The CMA and Trio ES tests were implemented. Trio ES ruled out other possible monogenic causes for the family. After reviewing cases and literature with duplications within this genomic region, we found that they are extremely rare and most of the cited cases were too small for comparison. The disturbance of the *BMP2* gene could explain BDD, but the other clinical presentations in the mother and fetus are not yet fully understood.

**Conclusion:**

This study provides important evidence for the current understanding of genotype–phenotype association of this 6.3 Mb size duplication in the 20p13p12.2 region. This duplication is a unique CNV occurring so far only in this family. Further cases and research are needed to understand the discordance in the phenotypes between the mother and fetus and establish the relationship between *BMP2* gene and BDD.

**Supplementary Information:**

The online version contains supplementary material available at 10.1186/s13039-022-00584-3.

## Introduction

The vast proportion of genetic diseases which can affect newborns can lead to early death, significant economic burden and can be overwhelming for the social medical system [1, 2]. Chromosomal microdeletions, microduplications and complex rearrangements causing severe birth defects account for an important part of etiology in genetic diseases [3–5]. Since the introduction of chromosomal microarray analysis (CMA), prenatal diagnosis can detect copy number variation (CNV) to unravel the genetic causes of fetal structural anomalies particularly [6–8]. By detecting chromosomal imbalances, microdeletions and microduplications, high density CMA can reveal step-by-step genotype–phenotype associations, expanding the current understanding of prenatal manifestations. Recently, exome sequencing (ES) has been implemented as a further diagnostic step in cases which were negative after karyotyping and CMA testing. The results showed that ES expand the diagnostic yield by as much as 32%, and it was especially efficient in fetal structural anomalies [9–11]. Therefore, the strategy of combining CMA and ES has become more commonplace in prenatal diagnosis.

Brachydactyly (BD) is a shortening of the fingers and toes and malformation of bones due to abnormal development of the phalanges and/or the metacarpals [12]. BD can occur, either as an isolated malformation, or as part of a complex malformation syndrome. Based on which bones are affected, different types of BD can be classified. The commonly used classification system of BD was first introduced by Bell et al., who classified BD into five main groups and several subtypes [13]. For instance, BD Type A (BDA) is characterized by the shortening of the middle phalanges, which is further subclassified by affected finger types, whereas BD Type D (BDD) is more common and only affects the thumbs. In type D patients, the distal phalanx of the thumbs is shortened unilaterally or bilaterally but all other fingers are normal. All types of BD, from Type A to E share some similar clinical features, such as hypoplasia of phalanges or interdigital joint malformation. Sometimes, a fetus with BD may have clinical manifestations which can be detected by ultrasound [14, 15]. Recently detailed guidelines have been proposed for prenatal diagnosis of BD [16]. However, further prenatal cases with a genetic diagnosis of BD are needed to fully understand its development.

Here, we report a maternally inherited microduplication of 20p13p12.2 (size 6,338 Mb) in a fetus. Both mother and fetus showed BDD. The duplicated region covered the *BMP2* gene, associated with BD type A2 (BDA2) (OMIM#112,600). The trio ES analysis did not reveal any other possible genetic causes. By presenting the clinical phenotypes and genetic results of both fetus and mother, we aim to present a genotype–phenotype association of this 6.3 Mb microduplication on chromosome 20. To gain a more comprehensive understanding of the variant’s pathogenicity and relationship with the clinical phenotypes, other similar cases and gene functions within this duplicated region were checked by literature review.

## Methods

### Patient samples

Patients’ family members signed an informed consent form. The fetus and family members were examined at the Fetal Medicine Unit and Prenatal diagnosis center, Shanghai First Maternity and Infant Hospital, Tongji University, Shanghai, P.R China. Here, we report a 29-year-old pregnant woman, non-consanguineous to her husband, who was given an intermediate risk (1/523) for Down syndrome in the second trimester maternal serum screening. Non-invasive prenatal testing at 20 weeks of gestation suspected fetal sex chromosome aneuploidy. An amniocentesis was performed at 22 weeks and 1 day of pregnancy. The couple claimed no family history of genetic diseases. There is no history of taking teratogens, irradiation, infections, smoking, diabetes, or hypertension during this pregnancy.

### Chromosomal microarray analysis (CMA)

Genomic DNA was extracted from 10 mL of uncultured amniocytes from the fetus and the peripheral blood of the parents using the QIAamp DNA Blood Mini kit (Qiagen, Valencia, CA, USA) following the manufacturer’s instructions. The fetal DNA was analyzed by Human CytoScan 750 K Array (Affymetrix Inc., Santa Clara, CA, USA). After comparing the size of duplicated region and cost for parental testing, the couple decided to first perform the mother’s CMA by the Human CytoScan Optima Array (Affymetrix Inc., Santa Clara, CA, USA). Knowing that the mother had a positive CMA result, the father only took part in the trio ES instead of CMA after financial concerns. Image data were analyzed using Chromosome Analysis Suite v4.0 software (Affymetrix Inc., Santa Clara, CA, USA). The results were analyzed using the Database of Chromosomal Imbalance and Phenotype in Humans using Ensembl Resources (DECIPHER), the Database of Genomic Variants, Online Mendelian Inheritance in Man (OMIM), and the National Center for Biotechnology Information.

### Trio exome sequencing (Trio ES)

Trio ES was performed with the fetal and parental DNA. The platform (NanoWES Human Exome, Berry Genomics, Beijing, China) was IIIumina NovaSeq 6000 (Illumina, San Diego, USA). The sequencing reads were aligned to the human reference genome (GRCh38). Verita Trekker® Variants Detection System by Berry Genomics and GATK version 4.0 software (https://software.broadinstitute.org/gatk/) were employed for variant calling, including checking single nucleotide variants (SNV) and small indels. CNV Variants were called by CNVkit software. All types of VCF files were annotated and filtered by Ingenuity Variant Analysis (https://variants.ingenuity.com). Common variants were filtered out using the Exome Aggregation Consortium (ExAC) (http://exac.broadinstitute.org), the Exome Sequencing Project (https://esp.gs.washington.edu), 1000 Genomes Project (http://www.1000genomes.org) and Genome Aggregation Database (http://gnomad-sg.org/) databases. Remaining phenotype-related variants were then assessed according to the protocol issued by the American College of Medical Genetics and Genomics/Association for Molecular Pathology (ACMG/AMP) guidelines [17]. Five well-established biological in silico prediction programs (SIFT, Polyphen2, LRT, Mutation Taster, and PhyloP) were used to predict the effect of missense variants. Human Splicing Finder (HSF) was used to predict the effect of splice site variants. All the selected variants were assessed for pathogenicity based on the adapted ACMG guidelines and the ClinGen sequence variant interpretation working group as well as updated recommendations for the ACMG criteria [18–20].

## Results

### Clinical manifestations

**Fig. 1:**
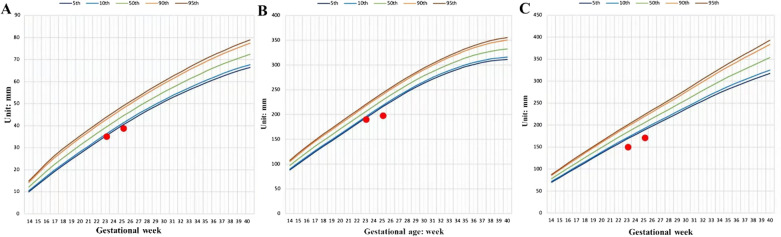
Growth parameters (red dots) on 23 + 1 weeks and 25 + 1 weeks were below the 5th percentile of the same gestational age, indicating a severe growth restriction for the fetus with a 6.338 Mb microdeletion on chromosome 20. **A** Fetal femur lengths. **B** Fetal head circumferences. **C** Fetal abdominal circumferences

The naturally conceived female fetus grew normally in the first trimester with nuchal translucency of 1.2 mm. A fetal structural screening ultrasound at 23 weeks and 1 day found no structural anomaly (digital details could not be observed due to the position), but a symmetric fetal growth restriction was noticed. The head circumference (HC), abdominal circumference (AC) and femur length (FL) were -2.83SD, -4.084SD and -1.84SD, respectively. The maximum vertical pocket (MVP) of amniotic fluid is 38 mm. Reduced fetal movement was also recorded. Follow-up fetal ultrasound at 25 weeks and 1 day showed persistent growth restriction with HC, AC and FL delayed to -4.119SD, -3.777SD and -2.376SD respectively (Fig. 1). The volume of the fetal bladder was significantly smaller than normal. The MVP of amniotic fluid decreased to 1.6 mm. However, the sizes and echogenicity were normal in the fetal kidneys and the placenta. The results of serial doppler assessments showed normal umbilical artery, ductus venosus doppler, middle cerebral artery and uterine artery flow. Maternal infection of cytomegalovirus, toxoplasmosis, rubella, or herpes were excluded. The mother had balanced nutritional intake and maternal hypertension, and diabetes were excluded.

**Fig. 2:**
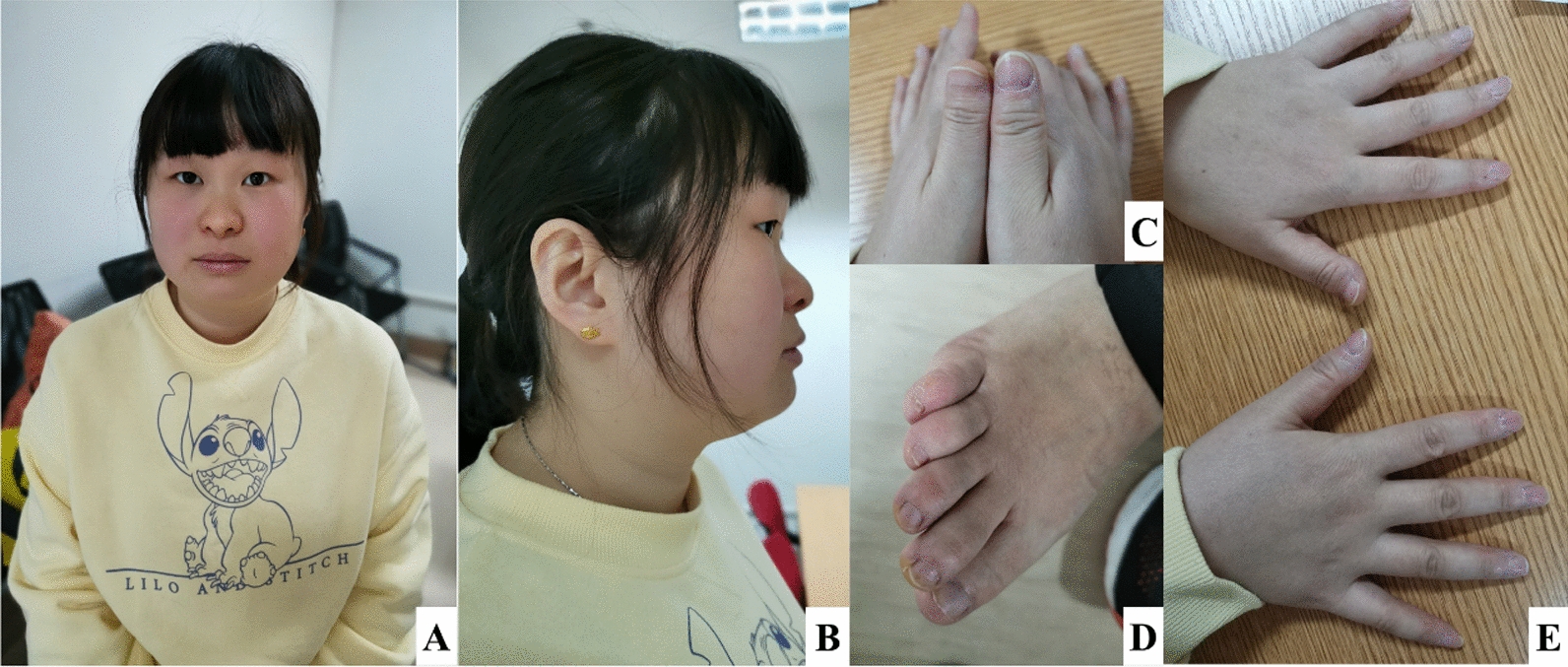
Facial dysmorphisms and brachydactyly in the mother with a 6.34cMb duplication (arr[hg38]20p13p12.2 (4,391,799–10,729,546) × 3). **A**, **B** midface hypoplasia, depressed nasal root, bulbous nasal tip. **C** Brachydactyly and nail hypoplasia on the distal phalange of left thumb. **D** Nail dysplasia on the distal phalange of the 5th right toe. **E** Overview of the comparison of both hands

The mother was born from a non-consanguineous Chinese Han couple from an uncomplicated natural pregnancy. She appropriately reached her developmental milestones. Growth delay, autism-spectrum disorders or behavior abnormalities were not present in her childhood. She has normal stature and shows no significant mental retardation. She responded appropriately during the conversation, the only abnormality being that she easily lost focus on conversion topics and showed difficulty in dealing with complex questions. She finished her education in middle school and had an academic performance of 20–30 points below average, in particular in mathematics and reasoning. Detailed physical examination showed that the mother had mild facial dysmorphisms with midface hypoplasia, depressed nasal root and bulbous nasal tip (Fig. 2A, B). Consistent with BDD, we noticed a unilateral BD on the distal phalange of left thumb in the mother and the nail of the thumb was flattened (Fig. 2C). The nail of the right 5th toe was hypoplastic (Fig. 2D). The other fingers were all normal (Fig. 2E). The cardiogram and renal echogenicity were normal.

**Fig. 3:**
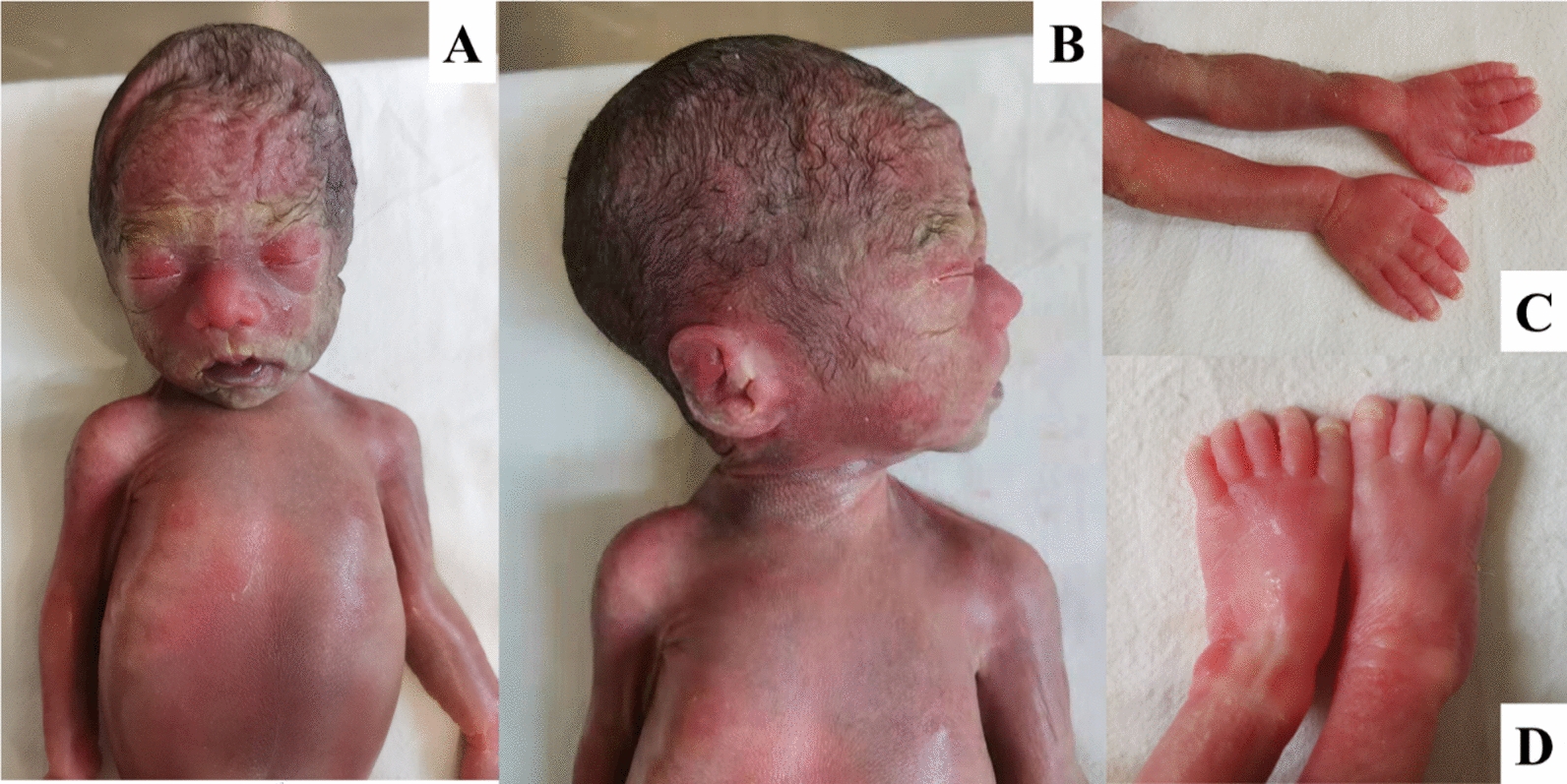
Facial dysmorphisms and brachydactyly of the terminated fetus. **A**, **B** midface hypoplasia, depressed nasal root, bulbous nasal tip, short philtrum, and posteriorly rotated ears. **C** Brachydactyly and nail hypoplasia on the distal phalange of left hallux. **D** Nail dysplasia on the distal phalange of the 5th right toe

After genetic counseling with regards to potential fetus phenotypes and the CMA and Trio ES results, the family decided to terminate the pregnancy at 26 weeks and 3 days. A 640 g demised female fetus was delivered. An autopsy was not performed on the terminated fetus as permission was declined by the parents. Postmortem external examination showed facial dysmorphism including depressed nasal root, bulbous nasal tip, midface hypoplasia, short philtrum, and low-set ears (Fig. 3A, B). The fetus also had BDD and hypoplastic nail on the distal phalange of the left hallux (Fig. 3C, D).

### CMA analysis

**Fig. 4:**
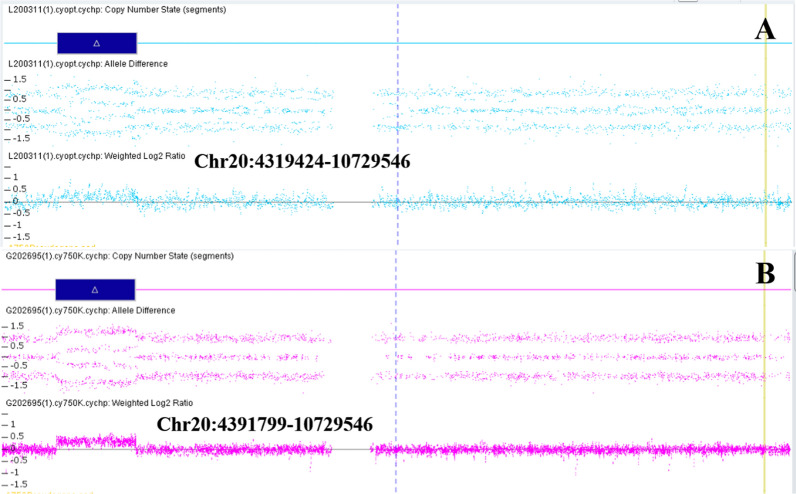
The identification of the duplication on chromosome 20 of the mother (**A**) and the fetus (**B**) by Chromosomal microarray analysis (CMA)

**Fig. 5:**
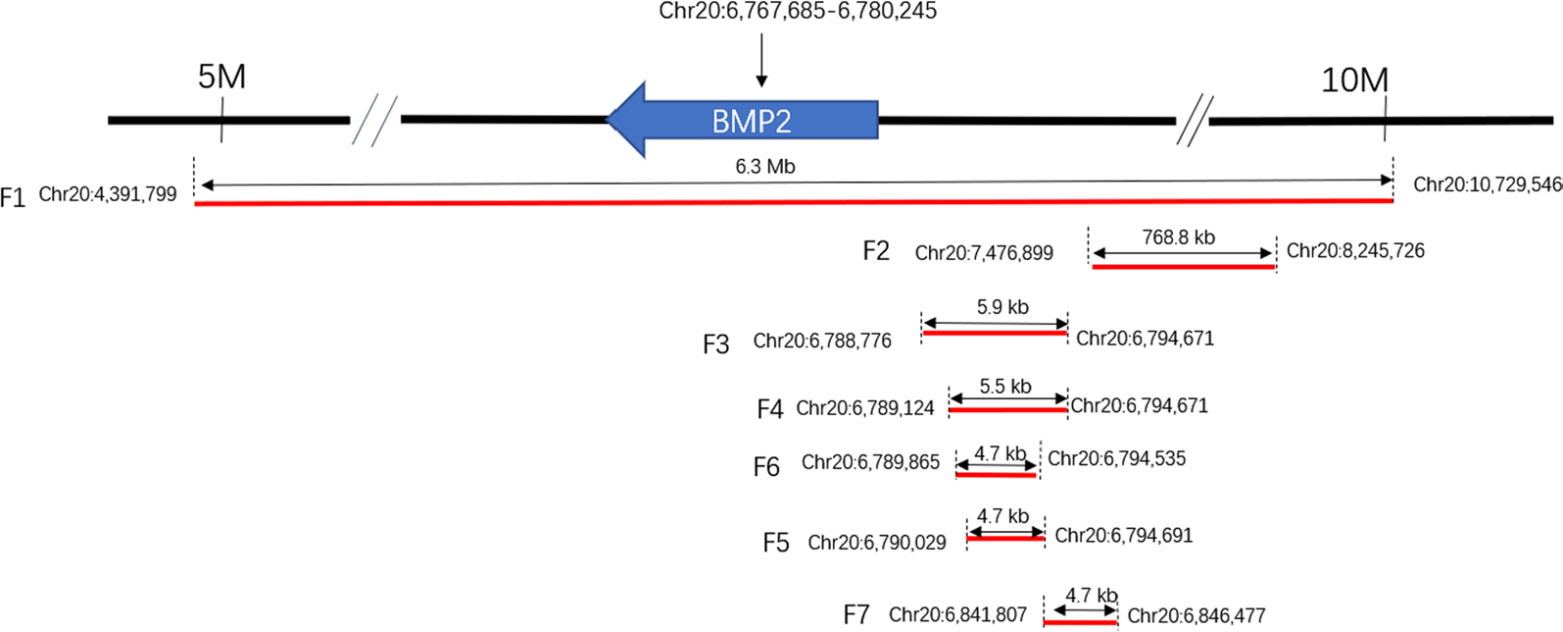
The comparison between the current microduplication and all other cited microduplication cases on chromosome 20 presented in Table 1

The SNP array showed a female fetus with a 6.34 Mb duplication (arr[hg38]20p13p12.2 (4,391,799–10,729,546) × 3) which was inherited from the mother (Fig. 4). This duplicated region covers eleven OMIM genes (https://www.omim.org/) including the *BMP2* gene (Table 1), related to BD type A2 (BDA2). One patient in Decipher (251,579) (https://decipher.sanger.ac.uk/) had a variant in a similar region, however no phenotype was recorded, and no record of other identical or similar CNV duplications has been found in public databases. To explore the relationship between genotype and phenotype, we checked the function and associated diseases with the genes within the 20p13p12.2 region (Table 1). By applying the current ACMG guidelines for the interpretation and classification of CNVs [21], the variant is classified as VUS because none of the genes contained within this duplication are located in a well-established triplosensitivity region. To address the pathogenicity of the CNV, we continued searching for other case studies concerning microduplications within or partially overlapping with our region (Table 2). In short, among the summarized cases, our case had the largest DNA fragment duplication (Fig. 5).

**Table 1:** Eleven OMIM documented genes and related diseases within the microduplication of 20p13p12.2 region

Gene	MIM Number	Related diseases	Inherited mode^#^
*PRNP*	176640	Creutzfeldt-Jakob Disease; CJD, Gerstmann-Straussler Disease; GSD, Kuru, susceptibility to, Insomnia, fatal familial, Huntington disease-like 1, Prion disease with protracted course	AD
*PCNA*	176740	?Ataxia-telangiectasia-like disorder 2	AR
*PROKR2*	607123	Hypogonadotropic hypogonadism 3 with or without anosmia	AD
*MCM8*	608187	?Premature ovarian failure 10	AR
*FERMT1*	607900	Kindler syndrome	AR
*BMP2*	112261	Brachydactyly, type A2, Hemochromatosis, Type 1; HFE1, Short stature, facial dysmorphism, and skeletal anomalies with or without cardiac anomalies	AD
*PLCB1*	607120	Developmental and epileptic encephalopathy 12	AR
*PLCB4*	600810	Auriculocondylar syndrome 2	AD/AR
*SNAP25*	600322	?Myasthenic syndrome, congenital, 18	AD
*MKKS*	604896	McKusick-Kaufman syndrome, Bardet-Biedl syndrome 6	AR
*JAG1*	601920	Alagille syndrome 1, Tetralogy of Fallot, Deafness, congenital heart defects, and posterior embryotoxon	AD

^#^Autosomal dominant (AD), Autosomal recessive (AR)

**Table 2:** Families with only microduplications of the 20p13p12.2 region, in particular duplications encompassing and in proximity to the *BMP2* gene

Families	Origins	Genes involved	Location on chromosome 20(hg38)	Size of duplicated region	Clinical phenotype	Reference(s)
Family 1	Chinese	*BMP2* and 10 more genes (see Table 1)	4,391,799–10,729,546	6.3 Mb	Unilateral thumb and hallux brachydactyly and hypoplastic nail	This study
Family 2	Caucasian	Noncoding region, downstream *BMP2, HAO1* and *TMX4,* and the first two exons of *PLCB1*	7,476,899–8,245,726	768.8 kb	Wolff–Parkinson–White (WPW) syndrome, no Brachydactyly	PMID:23239491
Family 3	Brazilian	Noncoding region, downstream *BMP2*	6,788,776–6,794,671	5.9 kb	Shortening of the second mesophalanx, a medial deviation in the proximal interphalangeal joint and absent of phalangeal flexion creases	PMID: 19327734 PMID: 7390514
Family 4	European	Noncoding region, downstream *BMP2*	6,789,124–6,794,671	5.5 kb	Hypoplastic, triangular middle phalanges	PMID: 19327734
Family 5	Chinese	Noncoding region, downstream *BMP2*	6,789,865–6,794,535	4.7 kb	Typical phenotypes of BDA2 characterized by medially deviated and shortened index fingers and second toes with abnormal interdigital joint formation	PMID: 24710560
Family 6	Chinese	Noncoding region, downstream *BMP2*	6,790,029–6,794,691	4.7 kb	Shortened, ulnar deviated, F2 malformed fingers and toes; F3 F4 syndactyl, variable phenotypes within the family	PMID: 21357617
Family 7	Chinese	Noncoding region, downstream *BMP2*	6,841,807–6,846,477	4.7 kb	Deviated, shortened index fingers with triangular-shaped middle phalanges, as well as in second toes, highly consistent within the family	PMID: 29129813

### Trio ES analysis

To exclude a monogenic disorder for the fetus, Trio ES was implemented.. The average sequencing coverage depth for the proband, mother and father was 74.7x, 102 × and 84.9x, respectively. The 20 × coverage for the proband, mother and father was 98%, 99% and 99%, respectively. Other detailed bioinformatic analysis quality indexes are shown in Additional file 1: Table S1.

After analyzing the sequencing data and filtering the variants, results did not show any pathogenic/likely pathogenic variants which can explain the phenotype in the fetus and mother. Special attention was given to BD related genes, for instance, the gene HOMEOBOX D13 (*HOXD13*; OMIM*142,989, sequencing coverage 100%), associated with BDD. We found only one maternally inherited VUS mutation in the *ARSE* gene (ChrX: 2,960,421, NM_000047.2:c.-20-1G > A(p?)), related to Chondrodysplasia punctata (OMIM#302,950). One hemizygous variant in the *ARSE* gene has been recorded in gnomAD (https://gnomad.broadinstitute.org). According to the ACMG guidelines, the classification of this variant is VUS. The clinical phenotypes of the fetus and mother partially overlap with Chondrodysplasia punctata’s manifestations, such as short stature and developmental delay. Finally, we did not report this variant because of the unsupported pathogenicity and the limited phenotypic overlap. The 20p13p12.2 microduplication detected by CMA was also confirmed by ES data (Additional file 1: Table S2; Additional file 2: Figure S1), however, due to the differences in the technique and platforms, the breakpoints of the duplication found by Trio ES test were slightly different when compared to the CMA results.

## Discussion

Here, we report a rare prenatal case of a maternally inherited 6.34 Mb duplication (arr[hg38]20p13p12.2(4,391,799–10,729,546) × 3) with BDD, severe prenatal intrauterine growth restriction and oligohydramnios. The mother also has BDD and exhibits further phenotypes of mild mental retardation and facial abnormality. This case contributes to the current understanding of genotype–phenotype association of patients with a duplication of the 20p13p12.2 region, which can facilitate future genetic diagnoses.

To explain the pathogenicity of the 6.34 Mb microduplication in our patients, we checked other prenatal diagnosis cases of CNVs of a similar length within and overlapping with this region. One prenatal diagnostic study described a fetus with a 5.28 Mb deletion at 2q37.3 and a 11.64 Mb duplication at 20p13p12.2 [22]. The fetal ultrasound results indicated intrauterine growth restriction, left kidney agenesis, right kidney dysplasia, ventricular septal defect, and polyhydramnios. The author speculated that the abnormal phenotype of the fetus may be due to both the deletion and duplication, which limits the reference value of this case. Another study described a 2-year-old patient who had a 5.37 Mb microdeletion at 20p13p12.2 (chr20:3,672,605–9,042,183). The patient had a cleft palate and facial dysmorphism [23]. Aside from this, the patient had neural muscular complications, such as delayed/poor reflexes, central hypotonia and decreased muscle mass. The author claimed that the *BMP2* gene in this region is a crucial factor for orofacial development and heterozygous loss of *BMP2* allele caused the resulting phenotype. Although the length and the region which was deleted described is nearly identical to our case, microdeletion and microduplication variants have an entirely different pathogenic mechanism. Following this, a focused study of the literature describing microduplications within this region with smaller size was performed (Table 2, Fig. 5). Duplicated sizes ranged from 4.7 kb to 768.8 kb and the patients’ phenotypic profiles varied, however, nearly all exhibited congenital malformation of fingers and toes [24–29]. Most of the reported CNVs are too small to compare with that of our patient. The CNV of our case appears to be a unique CNV occurring solely in this family, therefore, segregation of the variant with the disease in additional family members should be further evaluated to further elucidate the clinical significance of this CNV.

Until now, three genes, *BMP2, BMPR1B* and *GDF5,* have shown associations with BDA2 (OMIM#112,600). BDA2 follows an autosomal dominant mode of inheritance. The *BMP2* gene plays a central role in early embryogenesis, skeletal development, and differentiation of preosteoclasts into mature osteoblasts. It regulates bone morphogenetic proteins (BMPs), which are a group of growth factors belonging to the transforming growth factor beta superfamily [30]. BMPs combine and are activated by the bone morphogenetic receptor 1 (BMPR1) and bone morphogenetic receptor 2 (BMPR2) on the cell membrane in this signaling pathway [31]. The loss of function of the subgroup receptor BMPR1B, belonging to BMPR1, can lead to BDA1 or BDA2 [32]. Many other regulatory elements also play important roles in the BMP signaling pathway. For example, many BDA2 families were genetically diagnosed due to the duplication of the downstream enhancer region of the *BMP2* gene. A 5.5 kb region downstream of the *BMP2* gene was identified as a *cis*-acting enhancer element and was validated in the mouse model [25, 29]. Duplication CNVs of the *BMP2* gene as well as its up or downstream regulatory region have mostly been reported in BDA2 patients (Table 2). For instance, one study documented a 4.6 kb genomic duplication in the 20p12.2–12.3 region downstream of the *BMP2* gene associated with BDA2 in a Chinese family (Table 2) [28]. Most of the studies in Table 2 suggest that the duplication of the enhancer element downstream of *BMP2* gene can cause BDA2, with the exception of one case with Wolff–Parkinson–White (WPW) syndrome [24]. The author speculated that the main phenotype of WPW was caused by two well-characterized genes: *HAO1* and *TMX4*, and the first two exons of the *PLCB1* gene. So far BMP2 defects have been reported to be associated only with BDA2 (OMIM #112,600). Here, we speculate that the duplication of the *BMP2* gene region might also have caused BDD in both our fetus and mother based on the molecular function of *BMP2* gene.

To address the differing clinical phenotypes of the fetus and mother, we investigated the genes in the microduplication region further. In total 11 OMIM genes are involved (Table 1). Of these, five genes have autosomal dominant inheritance in this region of microduplication. With the exception of the *BMP2* gene, only the gene *SNAP25* (OMIM#600322), which is related to congenital Myasthenic syndrome 18, has been reported in prenatal diagnosis with decreased fetal movements and in utero onset for possible prenatal clinical diagnosis [33]. The remaining four autosomal dominant genes have not been reported in any prenatal cases upon reviewing current literature. The *PRNP* gene (OMIM# 176,640) is associated with various types of hereditary neurodegenerative spongiform encephalopathies, including Creutzfeldt-Jakob disease (CJD), Gerstmann-Straussler Disease (GSD), fatal familial insomnia (FFI) and Huntington disease-like 1. The *PROKR* gene (OMIM#607,123) associates with congenital idiopathic hypogonadotropic hypogonadism (IHH) which is characterized by absent or incomplete sexual maturation by the age of 18 years. The *PLCB4* gene (OMIM#600,810) is known to be associated with autosomal dominant craniofacial malformation syndrome. *JAG1* gene (OMIM#601,920) defects result in neonatal jaundice, cardiac disease, skeletal abnormalities, ocular abnormalities and Tetralogy of Fallot. A de novo 797 kb microduplication of 20p12.2 which included *JAG1* was reported in a 7-year-old girl who showed a wide range of symptoms including facial dysmorphism, intellectual disability, congenital heart defect and behavioral concerns, such as ADHD (Attention Deficit Hyperactivity Disorder), SPD (Sensory Processing Dysfunction), motor clumsiness, and poor self-regulation [34]. Despite having a much smaller duplicated segment than our patients, which included only the *JAG1* morbid gene and part of the non-morbid gene *SLX4IP*, the girl presents with more severe developmental and neurological phenotypes. The mother in our study has a normal cardiogram and no developmental delay and can express herself properly with no behavioral concerns. With current available evidence, the cause of these phenotypic differences could not be elucidated. Therefore, the 7-year-old girl may require further genetic testing, for instance ES, to exclude other possible genetic causes, such as point mutations in critical genes. Overall, we speculate that the extra copies of *PRNP*, *PROKR*, *PLCB4*, *SNAP25* and *JAG1* gene may not have gain-of-function dose pathogenicity in this family. Further research and more cases are needed to confirm this hypothesis.

The fetus in this report had early-onset severe intrauterine growth restriction with oligohydramnios which was detected at a gestational age of 23 weeks. Following the clinical guidelines of American College of Obstetrics and Gynecology [35], maternal diseases related to fetal growth restriction such as hypertension, autoimmune disease, antiphospholipid antibody syndrome, renal insufficiency, cardiac disorders, infection were all excluded. Postmortem autopsy would have unraveled additional phenotypes missed by routine ultrasonic scan, providing diagnostic information in both diagnosed and undiagnosed fetal abnormalities, in particular for the unexplained fetal hydrops and growth restriction. Up to 46.9% cases of internal examinations during fetal autopsies were reported to have had only internal malformations, which could assist genetic counseling regarding the recurrence risk [36]. However, in this study the couple declined a postmortem autopsy and magnetic resonance imaging (MRI) for ethical and religious reasons. Only external investigation and photography were consented to with the purpose of research and teaching. Aside from the 20p13p12.2 duplication, we could not find any other genetic alteration by CMA or Trio ES to explain the intrauterine growth restriction. The shape of placenta and umbilical insertion were also normal. Doppler velocimetry did not identify any abnormal blood flow waveform in the umbilical artery, the ductus venosus, middle cerebral artery or the precordial venous system. A potential placental insufficiency was less likely although could not be completely excluded as genetic testing of the placenta was declined.

The fetus and mother showed notable phenotypic differences that can be attributed to variable expressivity and reduced penetrance, which are commonly reported in microdeletion/duplication syndromes [37]. However, due to the limited number of patients in this family, we could not estimate the penetrance of 20p13 microduplications at this time. Another reason for the discordance among prenatal and postnatal phenotype was the scarcity of phenotype-genotype association studies for genetic disease during the prenatal period. We speculate that it is possible that the mother had a more similar or partially overlapping phenotype with the fetus during the prenatal period, which has not been recorded. In recent times, after prenatal ES was implemented into clinical practice, discordance has emerged between prenatal and postnatal phenotypes of genetic disorders [38–40]. For instance, the *SMAD3* gene was reported to cause prenatal agnathia-otocephaly complex in contrast to postnatal aortic aneurysm, cardiac anomalies, cleft palate and micro/retrognathia (Loeys-Dietz syndrome 3, OMIM#613795) [38]. The study also described one homozygous missense variant in gene *PIGW* which segregated in two sibling fetuses with prenatal Dandy-Walker malformation, hydronephrosis, hypoplastic kidney, genital hypoplasia and diaphragmatic hernia, overlapping with Fryns- or Fryns like syndrome [38]. In contrast, the postnatal cases with variants in *PIGW* were described clearly in public database to cause a glycosylphosphatidylinositol biosynthesis defect 11 (OMIM 610,275) presenting with developmental delay, intellectual disability, and seizures. Severe symmetrical intrauterine growth restriction with oligohydramnios of the fetus in our case is probably caused by the segmental effect of the 20p13p12.2 duplication. Further cases and functional evidence are required to clarify this association.

In conclusion, a female fetus with a maternally inherited microduplication of the 20p13p12.2 region is presented in this current study. Both fetus and mother were diagnosed with BDD. However, the fetus showed severe growth retardation, while the mother has mild facial dysmorphism and mild mental retardation. Our study showed that the duplication of the *BMP2* gene and its enhancer element region could cause BDD alongside with BDA2. Both CMA and Trio ES diagnostic approaches did not find any further genetic cause in addition to the microduplication of the 20p13p12.2 region. The *BMP2* duplication can partially explain the phenotype in this family, but the cause of the other clinical presentations of the mother and fetus are not yet fully understood, especially when considering the severity of the clinical phenotype in the fetus. Other than brachydactyly, the mother’s phenotype is quite mild when compared to that of the fetus. Variable expressivity and/or incomplete penetrance of the microduplication region still needs to be further investigated. Duplications in this region are extremely rare in the literature. The current CNV appears to be a unique CNV in this family, and therefore, additional family members need to be tested to further evaluate its clinical significance.

## Supplementary Information

**Additional file 1.** **Table S1**: The bioinformatic quality index of Trio ES of the fetus and mother and **Table S2**: The 6.3 Mb microduplication on chromosome 20 was detected by Trio ES data of the fetus and mother.

**Additional file 2.** **Figure S1**: The identification of the duplication on chromosome 20 of the fetus (A) and mother (B) by Trio ES data. The CNV kit software detected the duplicated region is seq[hg38] 20p13p12.2 (4,675,054-10,673,742)×3.

## Data Availability

The data and material that support the findings of this study are available from the corresponding author (LMS) upon reasonable request.

## Abbreviations

CMA: Chromosomal microarray analysis
ES: Exome sequencing
BDD: Brachydactyly type D
BDA2: Brachydactyly type A2
CNV: Copy number variations
BMPs: Bone morphogenetic proteins (BMPs)
WPW: Wolff–Parkinson–White
