# Supplementary material for: A 6.3 Mb maternally derived microduplication of 20p13p12.2 in a fetus with Brachydactyly type D and related literature review
Source: Mol Cytogenet. 2022 Feb 28;15:6. doi: 10.1186/s13039-022-00584-3 (PMC8887085; doi:10.1186/s13039-022-00584-3)

**Figure S1：**The identification of the duplication on chromosome 20 of the fetus (A) and mother (B) by Trio ES data. The CNV kit software detected the duplicated region is seq[hg38] 20p13p12.2 (4,675,054-10,673,742)×3.

**Figure S1**


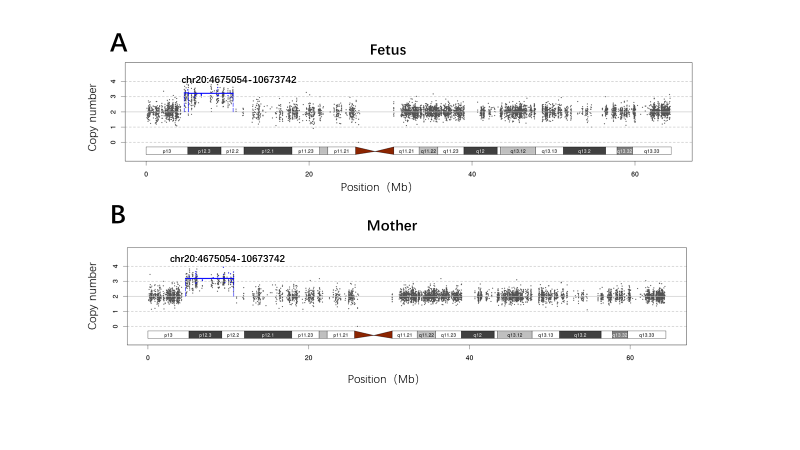

Supplement: Supplementary file 2 — Additional file 2. Figure S1: The identification of the duplication on chromosome 20 of the fetus (A) and mother (B) by Trio ES data. The CNV kit software detected the duplicated region is seq[hg38] 20p13p12.2 (4,675,054-10,673,742)×3. [file 13039_2022_584_MOESM2_ESM.docx]
